# New Medical Journals

**Published:** 1860-03

**Authors:** 


NEW MEDICAL JOURNALS.—It affords us pleasure to chronicle the advent of four new medical periodicals, whose titles are as follows:—

The *British American Medical Journal*, devoted to the advancement of the medical and physical sciences in the British American Provinces. Edited by Archibald Hall, M.D., F.R.C.S., Professor of Midwifery and the Diseases of Women and Children in the University of McGill College, Montreal. Montreal, January, 1860.

The *Chicago Medical Examiner*, a monthly journal, devoted to the educational, scientific, and practical interests of the medical profession. Edited by N. S. Davis, M.D., Professor of Medicine in the Lind University; and E. A. Steele, M.D. Chicago, January, 1860.

The *Kansas City Medical and Surgical Review*. Edited by G. M. B. Maughes, M.D., and T. S. Case, M.D. Kansas City, January, 1860.

The *Louisville Medical Journal*. Edited by Thomas W. Colescott, M.D. Louisville, February, 1860.

Here, then, are four new medical journals, and all born about the same time! We wish them all a long and prosperous career, hoping that the shadows of the editors, among whom we recognize some old and valued friends, may extend farther and farther until they cover the length and breadth of our own country and of the British Provinces of North America! Professor Hall, of Montreal, is well known to the profession of the United States as the able editor of a former medical journal, and for his valuable contributions to medical science. He is a gentleman of great practical ability, highly educated, and universally recognized as one of the leading obstetricians in Ca nada. Professor Davis is a veteran in journalism; and, with the aid of his colleague, Dr. Steele, the *Chicago Examiner* cannot fail to make its way to patronage and distinction. Drs. Maughes and Case have given evidence, in the first number of their *Review*, the only one that has reached us, of marked ability and good taste, and we wish them well in their new enterprise. Dr. Colescott was formerly associated with Dr. Drake and Dr. Yandell in editing the *Western Journal of Medicine and Surgery*, but abdicated long before the death of that periodical, in 1855. We hail his return to the editorial corps with much pleasure, convinced that he will conduct the *Louisville Medical Journal* with great ability and piquancy, and without any regard whatever to sectionalism, party feeling, college interests, or medical cliques. The whole number of medical periodicals at present in the United States must be about fifty, assuredly quite enough for all useful purposes. We shall be happy to place these new journals on our exchange list.

